# The Mechanism of Diarrhetic Shellfish Poisoning Toxin Production in *Prorocentrum* spp.: Physiological and Molecular Perspectives

**DOI:** 10.3390/toxins8100272

**Published:** 2016-09-22

**Authors:** Thomas Chun-Hung Lee, Fiona Long-Yan Fong, Kin-Chung Ho, Fred Wang-Fat Lee

**Affiliations:** School of Science and Technology, The Open University of Hong Kong, Hong Kong 852, China; chhlee@ouhk.edu.hk (T.C.-H.L.); lyfong@ouhk.edu.hk (F.L.-Y.F.); kcho@ouhk.edu.hk (K.-C.H.)

**Keywords:** diarrhetic shellfish poisoning, dinoflagellates, microalgae, okadaic acid, *Prorocentrum*

## Abstract

Diarrhetic shellfish poisoning (DSP) is a gastrointestinal disorder caused by the consumption of seafood contaminated with okadaic acid (OA) and dinophysistoxins (DTXs). OA and DTXs are potent inhibitors of protein phosphatases 2A, 1B, and 2B, which may promote cancer in the human digestive system. Their expression in dinoflagellates is strongly affected by nutritional and environmental factors. Studies have indicated that the level of these biotoxins is inversely associated with the growth of dinoflagellates at low concentrations of nitrogen or phosphorus, or at extreme temperature. However, the presence of leucine or glycerophosphate enhances both growth and cellular toxin level. Moreover, the presence of ammonia and incubation in continuous darkness do not favor the toxin production. Currently, studies on the mechanism of this biotoxin production are scant. Full genome sequencing of dinoflagellates is challenging because of the massive genomic size; however, current advanced molecular and omics technologies may provide valuable insight into the biotoxin production mechanism and novel research perspectives on microalgae. This review presents a comprehensive analysis on the effects of various nutritional and physical factors on the OA and DTX production in the DSP toxin-producing *Prorocentrum* spp. Moreover, the applications of the current molecular technologies in the study on the mechanism of DSP toxin production are discussed.

## 1. Introduction

Diarrhetic shellfish poisoning (DSP) has a worldwide incidence, and its occurrence has been recorded in Europe, Asia, North America, South Africa, Australia, and New Zealand since the 1960s [1,2,3,4,5,6,7]. It is an alimentary intoxication caused by a suite of DSP toxins produced by the dinoflagellates *Dinophysis* and *Prorocentrum* [5,8,9,10]. The expression levels of biotoxins in these dinoflagellates are strongly affected by nutritional and environmental factors [11,12,13,14,15,16,17,18,19,20,21,22,23,24]. People who consume shellfish containing bioaccumulated DSP toxins may present with non-fatal gastrointestinal symptoms, such as nausea, vomiting, abdominal pain, and most commonly, diarrhoea within 12 h of consumption [25], because of a water imbalance caused by the hyperphosphorylation of ion channels in the epithelial cells lining the intestine [26]. More than 200 people in Mainland China have recently shown symptoms of DSP after ingesting the contaminated mussels *Mytilus galloprovincialis* [5], with an okadaic acid (OA) concentration 40 times above the European Union regulatory limit of 160 μg OA equivalent/kg [27].

**Figure 1:**
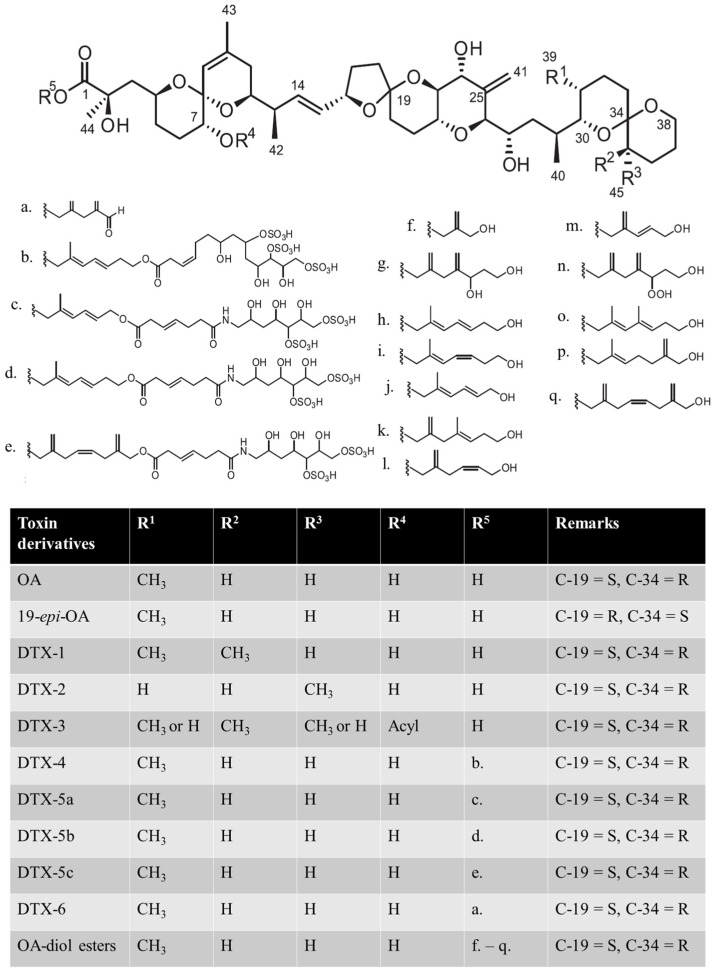
Structures of okadaic acid (OA), dinophysistoxins (DTXs), and their derivatives. C-19 and C-34 denote the 19th carbon and 34th carbon atoms, respectively; S and R denote the anticlockwise and clockwise stereochemistry of the carbon, respectively.

OA was first discovered and isolated from the marine black sponges *Halichondria okadai* [28] and *H. melanodocia* [29] in 1981. It is a globally distributed marine toxin and a main representative DSP toxin. Dinophysistoxins (DTXs), yessotoxins (YTXs), and pectenotoxins (PTXs) were also deemed DSP toxins; however, since 2002, YTXs and PTXs were not considered DSP toxins because they led to liver necrosis and cardiac muscle damage without diarrhoea symptoms [30]. OA and DTXs encompass several toxin derivatives (Figure 1); however, only OA and DTX analogues such as DTX-1, DTX-2, and “DTX-3” are the major toxins that induce intoxication [27]. “DTX-3” is a group of fatty acid ester derivatives caused by acylation of DTX-1 in the digestive gland of seafood [31]. Moreover, “DTX-3” metabolically transform back to DTX-1 in human stomach after consuming contaminated bivalves [32]. Therefore, “DTX-3” does not exist in microalgae [33]. These toxins are lipophilic and accumulate in shellfish and are potent inhibitors of serine/threonine protein phosphatases 2A (PP2A), 1B, and 2B [25], which are vital for the regulation of cell metabolism, DNA replication, transcription, RNA splicing, cell cycle progression, differentiation, and oncogenesis through the dephosphorylating phosphor-serine and phosphor-threonine residues of their substrates [34]; these toxins are potential tumor promoters [35,36,37] in the human digestive system [38]. Moreover, OA induces apoptosis [39,40,41,42,43,44], cytotoxicity [45,46], DNA adduct formation [47], chromosome loss [48], DNA breaks and cell cycle arrest [42], as well as changes in neuropeptide Y [49].

Among different shellfish poisoning toxin types, the effects of varied levels or components of paralytic shellfish poisoning (PSP) toxins in *Alexandrium* spp. under different environmental conditions have been studied extensively in the last twenty years [50,51,52,53,54,55,56,57,58]; however, a mechanistic study on DSP toxins has not been conducted. Moreover, most DSP toxin studies concern benthic *Prorocentrum* spp. [13,14,15,16,17,18,19,20,21,22,23,24,58,59,60,61]. Following the development of a 3-step feeding protocol for *Dinophysis* spp. in 2006 [62], researchers have begun investigating the properties of DSP toxin production in such species [63,64,65]. Nevertheless, the mechanism of DSP toxin production in *Dinophysis* and *Prorocentrum* requires further elucidation.

Here, we review studies focusing on the growth and cellular toxin levels of the DSP toxin-producing *Prorocentrum* spp. at a physiological and molecular level and its applications as well as studies providing insight into omics technologies and research perspectives.

## 2. Nutritional Factors

Nutrients are critical for the growth and survival of all microorganisms, including microalgae. Microalgal growth requires micronutrients, vitamins, chelators, and macronutrients, such as nitrogen and phosphorus. In seawater, the concentrations of nitrogen and phosphorus may not fulfill the demand of the algae, because their concentrations can vary with time under dynamic conditions. These variations induce certain physiological changes, particularly in algal growth and levels of toxin produced. Several algal culture media, such as f/2, L1, and K, are commonly used for *Prorocentrum* spp. cultivation. The growth rates, maximum cell densities, and cellular toxin levels of the algal cells may be affected by multinutrient interactions and the complex composition of the culturing media. For instance, the differences in the maximum cell densities of the *Prorocentrum* spp. (Table 1 and Table 2) could be considerable—approximately 5000–50,000 cells/mL. However, information on the effect of these interactions and the composition is scant. Therefore, a comparison of studies regarding the direct effects of the media on the growth and toxin levels of *Prorocentrum* spp. is difficult. Nevertheless, the effects of 2 essential nutrient sources—nitrogen, phosphorus, and a trace element, chelating reagents—have been analysed, and are discussed in this section.

### 2.1. Nitrogen

Nitrogen plays an essential role in microalgal metabolism because it is involved in the synthesis of different essential cellular components, such as proteins and chlorophyll [66,67]. Table 1 shows the growth and levels of cellular toxin (pg/cell) of *P. lima* under different concentrations of nitrogen. Most autotrophic algae, such as benthic *Prorocentrum* spp., can grow in routine culture media for microalgae (e.g., f/2, L1, and K, containing 882 μM nitrates and 36.3 μM phosphates). Supplying various concentrations of nitrogen normally affects the growth rate, maximum cell density, and toxin production in algal cells.

The results reported in various studies have shown discrepancies. Vanucci et al. and Zhong et al. have suggested that the nitrogen concentration is directly proportional to the maximum cell density of *P. lima*, but not to its growth rate [14,15]. When the concentration of nitrate decreased from 882 to 17.7 μM, the maximum cell density of *P. lima* decreased by 5 times [15]. However, Li et al. and McLachlan et al. have reported that the nitrogen concentration is directly proportional to both the maximum cell density and growth rate of *P. lima* [12,17]. Although Zhong et al. indicated that a drop in nitrogen concentration may reduce the maximum cell density of *P. lima* [14], the effect is not significant because of the narrow range of nitrogen concentrations (12–100 μM) selected.

In addition to the maximum cell density and growth rate, nitrogen concentration can also affect the cellular toxin content of *P. lima*. In routine culture medium, the levels of cellular toxins of *P. lima* are typically higher at the stationary growth phase than at the exponential growth phase [12,16,17]. However, the toxin levels increase in case of nitrogen deficiency; for instance, at 0 and 300 μM nitrate, the toxin levels were approximately 4 times higher than those at 1000 μM nitrate [17]. In addition, an approximate one-fold increment was observed in the cellular toxin levels of *P. lima* grown in a nitrogen-limiting culture with less than 882 μM nitrate [12,15,23]. Another study also reported higher-than-normal cellular toxin levels in *P. lima* grown at low nitrate concentrations (12–100 μM) [14].

In addition to limiting the concentration of nitrogen, McLachlan conducted a stepwise addition of nitrate (i.e., 30, 100, 300, 1000, and 3000 μM), which was added consecutively to the *P. lima* growth cultures at 10-day intervals. Enriched nitrogen caused a continuous growth of algal cells, but the cellular toxin levels remained relatively low (≤8 pg/cell) [17]. This result, however, cannot be compared with that obtained by Varkitzi et al. and Wang et al., who also varied the concentrations of phosphates [16,22].

Several reports have shown that benthic *Prorocentrum* spp. can use different nitrogen sources such as ammonium, nitrate, urea, and amino acids [14,16,61,68,69,70] through various pathways [71]. *P.* *hoffmannianum* and *P. lima* first use ammonia when maintained in f/2 and L1 media, respectively, both with additional 50 μM ammonia [61,70], suggesting that ammonia may be a preferred nitrogen source for benthic *Prorocentrum* spp. Ammonia is a unique nitrogen source for phytoplankton because it does not require enzymatic fixation, and can simply be assimilated as amino acids via the glutamine synthetase/glutamate oxoglutarate aminotransferase (GS/GOGAT) pathway [71]. However, ammonia uptake may not favour the synthesis of DSP toxins because the uptake apparently does not raise cellular toxin levels [14,16,61]. Nitrate, however, must be reduced to ammonia by nitrate reductase before being assimilated as amino acids via the GS/GOGAT pathway; this reduction may provide additional phosphorylated high-energy compounds for toxin synthesis [71].

Amino acid uptake may also influence the growth and cellular toxicity of dinoflagellates. *Proroecntrum* *lima* can grow in an enriched K medium with 670 μM L-amino acids—serine, lysine, threonine, valine, leucine, and aspartic acid—with approximately 25% increments in maximum cell density in the presence of leucine and onefold increments in cellular toxin levels [69]. However, further investigation is required to investigate the role of amino acid in the formation of toxin.

### 2.2. Phosphorus

Phosphorus is a macronutrient involved in the catabolism of sugars and fatty acids and in cellular coordination; it is also a major component of membrane lipids and adenosine triphosphates (ATPs), DNA. The phosphorus concentration in a medium can influence the growth and toxin production of microalgae because it alters protein phosphorylation within the cells [72]. Similar to nitrogen, phosphorus concentration is directly proportional to the maximum cell density of *P. lima*, but there is a discrepancy in growth rate (Table 2) [13,15,19]. When the phosphorus concentration is lower than 30 μM, cellular toxin levels are high but not in direct proportionality (Table 2) [13,15,23]. However, no study has shown the effect of phosphate depletion on the growth and levels of cellular toxins in the DSP toxin-producing *Prorocentrum* spp.

Microalgae can use both inorganic phosphates—metaphosphates, pyrophosphates, tripolyphosphates, and orthophosphates—and organic phosphates—sugar, phospholipid, and nucleotide phosphates—from different sources [54]. Sodium dihydrogen orthophosphate uptake usually occurs before [15,16,70] or along with nitrate uptake [61,73] in *P. lima* and *P. hoffmannianum*. The uptake of phosphorus from different sources can affect the maximum cell density and growth rate of *P. lima* (Table 2). The maximum cell density of *P. lima* supplied with organic phosphates is usually higher than that of *P. lima* supplied with inorganic phosphates [13,18]. The maximum cell density of *P. lima* increases, but its growth rate decreases in the presence of 10 μM glycerophosphate [18]. In a similar manner, the maximum cell density of *P. lima* increases in the presence of ATPs [13]. 

*P.* *lima* obtains phosphorus from sodium dihydrogen orthophosphate, glycerophosphate, and ATPs, and shows different levels of cellular toxin. Among these 3 phosphate sources, the cellular toxin levels of *P.* *lima* were relatively higher in the presence of glycerophosphate [13]. Besides, the levels of alkaline phosphatase and hydrolytic activity in microalgae increase from exponential to stationary growth phases in the presence of glycerophosphates [18]. This may be attributed to the phosphorus uptake requiring hydrolysis of glycerophospate into glycerol and phosphate by membrane-bound alkaline phosphatase [74], suggesting that the remained glycerol may provide an additional carbon for enhancement of cellular toxin level.

### 2.3. Chelating Reagents

Chelating reagents stabilise the concentrations of free metal ions to a non-toxic level, and chelate iron to increase solubility [75] as well as regulate the growth and levels of cellular toxin in *P. lima*. For instance, Sohet et al. demonstrated that cells grown in humic acid have a shorter lag phase and lower cellular toxin levels, but a higher maximum cell density compared with those grown in EDTA; in addition, this effect is independent of the humic acid concentration [19]. Humic acid is a chelating reagent that enhances the availability of certain metal ions, such as iron and manganese ions, but reduces the toxicity of copper ions to algal cells [19]. Metal ions may act as co-factor of enzymes, promote their production and activity, and facilitate membrane permeability. In addition to humic acid, fulvic acid, soil extracts, and algal extracts are potentially beneficial chelators [76].

## 3. Physical Factors

Other than nutritional factors, algal blooms may be affected by physical factors such as the temperature, salinity, light intensity, light–dark cycles, and water turbulence. However, the studies of the effect of these physical factors are very limited. Among these factors, only temperature, salinity and light have been studied [17,20,21,22,24,59,60].

### 3.1. Temperature

Benthic *Prorocentrum* spp. are often present in tropical regions—such as the Caribbean, India Ocean, the tropical region of the Atlantic and the Pacific Ocean—where the water temperature is approximately 30 °C [77]. Different epiphytic *Prorocentrum* spp. are present in the North Aegean coastline of Greece, where the water temperature ranges from 10 to 29.5 °C [78,79]. However, a *P. lima* strain can also be found in the colder waters of the Sanriku coast in Northern Japan [80].

DSP toxin-producing *Prorocentrum* can tolerate a wide range of temperatures, depending on the original geographic location (Table 3) [17,20,21,59,60]. For example, *P. lima* isolated from the mid-temperate area of Nova Scotia in Canada can survive at 0 °C for 28 days [17] and grow between 5 and 25 °C [20]; however, if it is isolated from tropical regions, such as Knights Key in Florida, the United States, it can grow between 19 and 33 °C, but it cannot be revived after being incubated at 19 °C or below [59,60]. In a similar manner, *P. hoffmannianum* can tolerate temperatures between 21 and 36 °C, with its optimal growth observed at approximately 29 °C, depending on the light intensity [21]. *P.* *concavum* can grow between 21 and 31 °C, with its optimal temperature being 27 °C [21]. *P. Belizeanum* exhibit the most favourable growth at 25 °C, but the cells undergo thermal stress at 28 °C [24]. According to the data shown in Table 3, the optimal temperature for the growth of both *P. lima* and *P. belizeanum* is 25 °C [20,22,24,60]. *P.* *concavum* *and P. hoffmannianum* grow optimally at relatively high temperatures—27 °C [60] and 29 °C [21], respectively.

The temperature influences the growth rate, maximum cell density, and cellular toxin levels of *P. lima* [20] and *P. hoffmannianum* [21]; this may be attributed to the effect of the temperature on cellular metabolic and enzymatic activities (e.g., alkaline phosphatase activity is affected by temperature change) [59]. Both the growth rate and maximum cell density of *P. lima* appear to be directly proportionate to the temperature within 25 °C. Inhibition of growth at 30 °C has been reported. The cellular toxin level shows an increment in extreme temperatures. For instance, the cellular toxin levels of *Prorocentrum* under 5 °C are approximately 2–6 times higher than those at other higher temperatures [20]. The highest cellular toxin levels at low temperatures, 23 and 18 °C, have also been observed in *P. hoffmannianum* [21] and *P. belizeanum* [24], respectively. Recently, the cellular level of OA and DTX-1 of *P. lima* increased by ~5 times and ~3 times in low temperature, 15 °C and even ~12 times and ~3 times in high temperature, 30 °C [22]. However, the relationship of the temperature on the changes in growth and cellular toxin levels remains unclear. Therefore, a conclusion cannot be drawn until additional studies are conducted on these species.

### 3.2. Salinity

Salinity is another critical physical factor affecting the growth rate of microalgae. *P. lima* and *P. concavum* can tolerate a salinity of 20–45 ppt and 20–43 ppt respectively, with an optimal salinity of 30 ppt; furthermore, *P. hoffmannianum* can tolerate a salinity of 28–40 ppt, with an optimal salinity of 34 ppt [21,22,59,60]. Certain *P. lima* strains can even grow at a salinity of higher than 40 ppt, such as in the mangrove root in Florida Keys, the United States [59].

In addition to the growth rate, salinity may also affect the cellular toxin levels of microalgae. The cellular OA level of *P. hoffmannianum* is the highest, at 28 ppt salinity (7 pg/cell), but it is the lowest at 34 ppt (approximately 3 pg/cell), with the salinity resulting in the most favourable growth of *P. hoffmannianum* [21]. However, *P. lima* showed a contradictory result. The highest cellular toxin level is at 45 ppt (OA: about 4 pg/cell, DTX-1: about 23 pg/cell) but the lowest level is at 15ppt (OA: about 0.5 pg/cell, DTX-1: about 4 pg/cell) [22]. Further study is required in order to confirm trend of cellular toxicity under different salinity.

### 3.3. Light

Light is an essential factor for all photosynthetic organisms to produce energy for survival and growth. Excessive light intensity may lead to photoinhibition in dinoflagellates [24,81]. For a better comparison, all of the irradiances described in this section are assumed to be sunlight at midday during summer in the 400–700-nm waveband and is expressed as luminance (lux), according to Woodward’s method [82]. In Morton’s study, the *P. lima* strain PL100A isolated from Knights Key in Florida, the United States, exhibits maximal growth at approximately 4000–4500 μW/cm^2^ (approximately 12297–13834 lux) [59,60], whereas *P. concavum* can grow at 1500 μW/cm^2^ (approximately 4611 lux) and reach maximal growth at approximately 5500 μW/cm^2^ (approximately 16908 lux). The maximal growth rate of *P. hoffmannianum* was found to increase from approximately 0.2 divisions per day (div/d) to 0.53 div/d when the luminance increased from 2000 to 5000 lux; however, this experiment was conducted under the influence of both light and temperature changes (Table 3) [21]. Furthermore, the growth rate of the *P. belizeanum* strain VGO1029 increased from approximately 0.1725 to 0.205 div/d when the irradiance increased from 40 µE/m^2^/s (approximately 52328 lux) to 80 µE/m^2^/s (approximately 104657 lux) at 24 °C. However, when the light intensity was excessive (i.e., 80 µE/m^2^/s (approximately 104657 lux)), the growth rate decreased to 0.12 div/d because of photoinhibition [24]. DSP toxin-producing *Prorocentrum* spp. typically appear to grow suitably under a wide range of light intensities (2000–104,657 lux). 

Light is an essential factor in DSP toxin production. Higher cellular toxin levels have been observed in lower light intensity conditions. Morton found a relatively higher cellular concentration of OA (53.75 pg/cell) at a lower light intensity (approximately 2000 lux) in *P. hoffmannianum* [21]. In a similar manner, a relatively higher cellular concentration of OA (approximately 4 pg/cell) was reported in *P. belizeanum* when the cells were grown under a lower-irradiance condition (20 µE/m^2^/s or 26,164 lux) [24].

Pan et al. indicated that toxin biosynthesis depends on the cell cycle of microalgae, which is controlled by light [61]. First, this study have shown that the cellular toxin amount per cell of OA, OA C8-diol-ester, DTX-1, and DTX-4 in *P. lima* remained unchanged when the change of cell cycle is paused by after dark acclimation [61]. After that, DTX-4 is synthesised from G1 to S phases in the morning, whereas OA is synthesised from S to G2 phases in the afternoon [61]. Based on the sequential increases in the levels of DTX-4, the OA C8-diol-ester, and OA, DTX-4 is transformed to the OA-diol ester, and then to OA through stepwise enzymatic reactions. The DTX-4 transport between cellular compartments is probably controlled through a light-mediated mechanism if the enzyme is compartmentalised from the DTX-4 synthesis sites [61].

In addition to light intensity, their wavelength also regulates the growth of photosynthetic organisms. Different algae have different optimal wavelengths for absorbing light energy. To absorb energy, most algae use chlorophyll a and carotenoids, whereas green algae use chlorophyll b and diatoms and brown algae use chlorophyll c [83]. Most of the dinoflagellates possess both peridinin and chlorophyll a to absorb light energy [84]. This may influence the cellular physiology of the algae. *P. Lima* has the most favourable cell division rate (0.58 div/d) at a wavelength similar to that of light in shallow oceanic water (450 nm) [59]. This finding is consistent with mangroves and lagoon habitats, which have abundant benthic *Prorocentrum* spp. and match the optimal wavelength of peridinin-chlorophyll a protein. However, to date, the correlation between cellular toxin levels and wavelengths remains unknown.

Photoperiods also influences the growth and cellular toxin level of *P. lima*. Wang reported that the light duration is direct proportional to maximum cell density. When the light duration increased from 8 h to 12 h or above, the maximum cell density increased by 1.5 times [22]. However, the optimal cellular OA and DTX-1 levels were noted at photoperiod (12 h:12 h), indicating that both photosynthesis and dark respiration is necessary for DSP toxin biosynthesis.

## 4. Bacteria Interaction

Interactions between the levels of cellular toxin of benthic *Prorocentrum* spp. and their associated bacterial microflora have been investigated: extracellular-attached bacteria, extracellular free-living bacteria, and intracellular bacteria can be observed through scanning and transmission electron microscopy [85]. Extracellular-attached *Roseobacter* sp. was reported in an ultrastructure study of a xenic *P. lima* strain [86,87]. Rausch de Traubenberg found that intracellular, extracellular-attached, and extracellular free-living alphaproteobacteria contain low quantities (approximately 1% of the total amount of toxin) of OA and DTXs [88]. The author suggested that this may be due to the adsorption of OA released from *P. lima* cells or toxin production of bacteria themselves. The role of these bacteria and their relationship with *Prorocentrum* cells is unclear, although it is suggested that they exert a synergistic effect on *P. lima* cells. In the senescence phase of *P. lima*, the growth conditions become unfavourable, and the levels of toxins increase. These bacteria possibly begin degrading the *P. lima* cells, accelerating the senescence of the microalgae. Lytic action may increase the quantity of dissolved organic carbon, which may provide more resources for enhancing toxin production [88].

Immunological studies have shown that toxin synthesis occurs in microalgae, rather than in the associated bacteria [10,89]. An Immunogold-labelling study involving the use of an OA antibody showed that most OA in *P. lima* and *P. maculosum* is located in the chloroplasts, whereas the lysosomes contain less OA [10]. OA is also observed at the cellular periphery, near the peripheral chloroplasts and variably sized vacuoles on the cytoplasmic periphery, as demonstrated using a polyclonal fluorescein isothiocyanate-labelled IgG antibody [89]. Moreover, Morton found that the levels of cellular toxins of xenic and axenic *P. hoffmannianum* do not differ significantly [21], suggesting that DSP toxins are originally produced by the microalgal cells, and not the associated bacterial cells.

## 5. Application of Molecular Tools for Studying the Mechanism of Algal Toxin Production

Omics technologies have been extensively applied in the life science fields, such as evolution, genetics, as well as developmental and cellular biology. Researchers have recently studied the mechanism of toxin synthesis from gene expression to protein levels by using omics technologies; however, the mechanism of toxin production in microalgae, including DSP toxin production, remains largely unknown. 

### 5.1. Genomics and Transcriptomics Studies

Full genome sequencing, an omics technology, determines the complete DNA sequences of target organisms including chromosomal, mitochondrial, and chloroplast DNA. Dinoflagellates contain a considerable amount of DNA (3 to 250 pg/cell) and chromosomes (20 to 325 per cell) [90,91]. If the amount of DNA is expressed in base pairs (bp), the estimated size of a dinoflagellate genome ranges from 3000 to 215,000 megabase pair (Mbp) [92], which is approximately 100 times larger than the human genome (3.2 pg DNA per cell and 3180 Mbp in 23 pairs of chromosomes); thus, the full genome sequencing of dinoflagellates, such as DSP toxin-producing *Prorocentrum*, may be impractical. McLean provided a comprehensive review of the application of omics in harmful algae. Omics analysis on the harmful algal species with a completed genome (e.g., domoic acid-producing species *Pseudo-nitzschia* *multiseries*) may provide extremely useful hints regarding other algal toxin production mechanisms [93].

In addition to DNA, investigations can also be conducted by isolating and expressing RNA transcripts of target organisms. Transcriptomics studies are technically less difficult compared with full genome sequencing studies because only a relatively smaller amount of complementary DNA (cDNA) is obtained using reverse transcription. Expressed sequence tag (EST) libraries have been constructed for identifying gene transcripts. A comparison of gene expression between toxic and non-toxic strains may enable the study of the physiological changes in microalgae during different growth phases and under various environmental and nutritional conditions [94,95,96]. 

The expression of translation-associated genes, intracellular signalling and selfish genetic element genes in dinflagellates can be characterised using microarrays to study the genetic properties and alterations in gene expression under nutritional and environmental stress [95,97,98,99,100,101,102,103,104,105].

Serial analysis of gene expression (SAGE) and massively parallel signature sequencing (MPSS) are technologies that can sequence and determine gene expression simultaneously [106,107]. Although this method can be conducted even without sequence information [108], a large amount of mRNA is required. MPSS is similar to SAGE, but has a relatively high sensitivity to examine rare transcripts in other dinoflagellates [108,109,110,111].

By using the current powerful next-generation sequencing (NGS), comparative transcriptomics studies on the toxic dinoflagellates have become more feasible and cost-effective [100,112,113]. Although these powerful technologies have been used extensively in the past few years, no transcriptomics study has been conducted on DSP toxin-producing *Prorocentrum* spp. Only a RNA sequence library of the *P. lima* strain (CCMP684) has been compiled by Keeling during a marine microbial eukaryote transcriptome sequencing project organised by the National Center for Genome Resources and the Gordon and Betty Moore Foundation’s Marine Microbiology Initiative.

Transcriptomics involves an advanced approach, and it has certain drawbacks. Because of the lack of complete genome data, only a limited amount of expressed genes can be identified (e.g., 27%–28% in *A. minutum* [94,96], 15% in *A. ostenfeldii* [100], 25% in *A. catenella* [101], 20% in *A. tamarense* [103], and 29% in *K. brevis*) [108]. In addition, dinoflagellates usually regulate RNA splicing and may affect the constitution and expression of RNA [92,114,115,116,117]. Moreover, the gene expression of dinoflagellates may be inconsistent with their protein expression. For instance, the protein levels of nitrogen-associated protein (NAP50) and Rubisco II, but not their corresponding gene expression, are strongly associated with the availability of nitrogen in *A. affine* cultures [118].

### 5.2. Proteomics

Proteomics is the large-scale study of structures, functions, and expression of proteins. This approach does not depend on the constituency and expression dynamics of transcriptomes. Potential target proteins can be screened through the comparative proteomic approach, usually in a gel- or non–gel-based manner. Classical gel-based comparative proteomics is used for separating protein mixtures by two-dimensional gel electrophoresis (2-DE), followed by mass spectrometry (MS) identification, such as matrix-assisted laser desorption ionisation–time of flight–mass spectrometry (MALDI–TOF–MS). By contrast, in non–gel-based proteomics, labelled and unlabelled proteins are digested using enzymes, and their relative abundance and identities are determined through multi-dimensional liquid chromatography coupled with electrospray ionisation tandem mass spectrometry (MS/MS) or MALDI–TOF–MS/MS. 

The 2-DE remains a powerful and widely used method for analysing complex protein mixtures extracted from cells, particularly those of non-model organisms, at a relatively lower cost compared with the non–gel-based method. It provides a fast overview of proteomes of interest and enables the detection of proteins with post-translational modifications (PTMs). In addition, the isoelectric point and molecular masses of intact proteins can be observed easily from the gel physically. The 2-DE can be analytical and preparative, and it can provide information regarding the expression and PTMs of proteins and allow the isolation of proteins at a significant amount (even up to milligram levels if required) for downstream structural analysis or de novo sequencing through MS/MS or Edman degradation. It is still commonly used because of the limitations of non–gel-based methods. For example, with the metabolic isotope-labelling method, the uptake of labelled amino acids may influence the cellular physiology, such as by enhancing the levels of cellular toxins present in dinoflagellates, including *P. lima* [69]. Furthermore, complete genome information may be required beforehand because the isotope-coded affinity tags have to ligate the cysteine residues of proteins, and it is also a prerequisite for the identification of differentially expressed proteins.

To identify differentially expressed proteins, a high-quality 2-DE gel with well-resolved protein spots is required. Protein extraction from dinoflagellates is a critical factor ensuring well-resolved protein spots. Lee and Lo successfully performed high-quality 2-DE for dinoflagellate samples by using a TRIzol reagent for protein extraction, and suggested a few benefits of its use [119]. First, the TRIzol reagent increased the simplicity and speed of nucleic acid and protein extraction. Second, the TRIzol reagent contains guanidine isothiocyanate, which denatures proteases to prevent protein degradation and enhances sample recovery without requiring the addition of other protease inhibitors [119]. In addition, RNA and DNA can be extracted simultaneously from the same sample for further analysis. This method has been widely applied in many dinoflagellate species, such as planktonic *P. donghaiense* [120], PSP toxin-producing *A. catenella* [121,122,123,124], and YTX-producing *Lingulodinium polyedrum* [125]. Nevertheless, a few reports have suggested that this method may not produce high-quality results for certain dinoflagellate samples. For example, Wang et al. indicated that quality of the protein spots of *A. catenella* strains obtained through TRIzol extraction may not always be promising [126]. Moreover, for unknown reasons, massive interfering substances appeared on the gel image of *P. hoffmannianum* samples [127]; these imply that protein preparation with a TRIzol reagent may not be universal to all types of dinoflagellates, and thus, slight modifications of the method may be required for particular species.

Proteomic studies on DSP toxin production are limited, and thus, finding the relevant literature is extremely difficult. Most proteomic studies in the area of algal toxins have focused on PSP toxin biosynthesis in *Alexandrium* spp. [118,122,123,124,128,129,130,131]. Certain researchers have compared the 2-DE profiles of 2 strains with considerable variations in the levels of toxins triggered by different environmental conditions or growth phases [124,129,130]. Others have examined differentially expressed proteins, which were observed by comparing the 2-DE profiles of 2 strains with dramatic variations in the levels of toxin [123,128]. Except *Alexandrium* spp., a phosphoamino acid-binding Pro-Q diamond stain was applied in the examination of rhythmic changes of phosphoproteome in *Lingulodinium polyedrum* to detect changes in protein phosphorylation after 2-DE [125]. Lacking sufficient DNA sequence information hinders protein identification in dinoflagellates. The construction of an EST library may be an approach that can improve protein identification. The study of *L. polyedrum* is an example of using this approach to optimise protein identification [125]. If the protein remains unidentified, it may be a novel protein. Lee et al. found the presence of NAP50 in *A. affine* cells without any genome information. The de novo partial peptide sequences were derived through LC–MS/MS of tryspin digested protein spot of NAP50. With the aid of molecular biology techniques, such as 5′ and 3′ rapid amplification of cDNA ends, the amino acid sequence of the open-reading frame was then deduced from the corresponding cDNA [118].

## 6. Putative Mechanism of DSP Toxin Production

The characteristics of DSP toxin production in *Prorocentrum* spp. and recent omics technologies on dinoflagellates were reviewed in the previous sections. Because of limited studies and the lack of genome information, the mechanism of toxin biosynthesis has not yet been completely elucidated. However, the chemical structures of toxins can provide some indication regarding their production. OA and DTXs are polyketide toxins that share a common structure of these metabolites—cyclic polyether. Snyder et al. reported that *P. lima* contains *PKSI* and *PKSII*, whereas *P. hoffmanianum* contains only *PKSI*, indicating that DSP toxins production may be strongly associated with PKS [132]. The *PKSI* sequence of *P. lima* has been determined, and the translated amino-acid sequence appears to be 99% similar to that of *P. micans* [133]. Moreover, PKS of *P. lima* and *P. mican* belongs to the same clade as that of *Nostoc punctiforme* in a neighbour-joining phylogenetic tree based on amino-acid sequences [133]. *N.* *punctiforme*, a cyanobacterium, is a microsytin producer [134]. Microsytin production is associated with the hybrid enzyme of PKS and non-ribosomal peptide synthase (hybrid NRPS/PKS) [135,136]. Therefore, PKS may be closely associated in DSP toxin production. 

Several precursor incorporation studies have also indicated that the PKS pathway participates in DSP toxin biosynthesis [137,138,139,140,141]. Glycolate from photorespiration is a deemed precursor of OA synthesis and side chains of DTX-5 (side chain c and d of R^5^ in Figure 1). They are formed through the typical polyketide process, the consecutive addition of acetate and interruption by Favorskii-like rearrangement [138]. The side chains of DTX-5 ligate OA through an ester bond between glycolate-derived hydroxyl group and terminal carboxyl group of OA [140]. In addition, amide in the side chain of DTXs may be replaced by glycine. After the incorporation of amino acids as precursors, chain termination or the midchain extension of units is usually directed by hybrid NRPS/PKS in cyanobacteria [136,142] and actinomycetes [142], suggesting that OA and DTXs are generated by hybrid NRPS/PKS [138].

**Figure 2:**
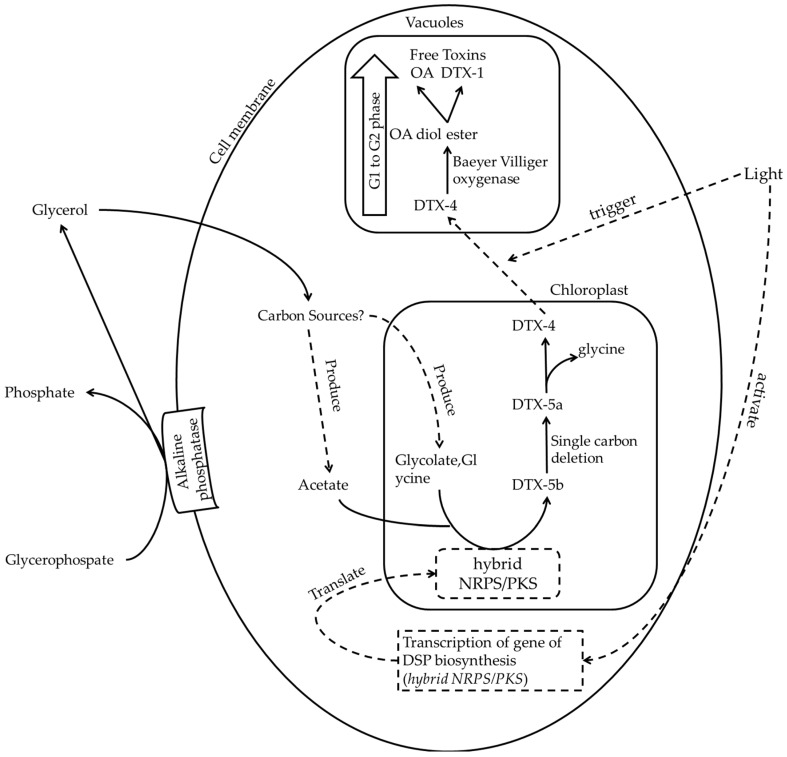
The postulated pathway of DSP toxin production in relation to NRPS/PKS. Light may trigger gene transcription for hybrid enzymes of non-ribosomal peptide synthase and polykeitide synthase (hybrid NRPS/PKS). The enzymes are then translated to produce DSP toxin. Acetate reacts with glycolate to form DTX-5b, which may undergo single carbon deletion to form DTX-5a. DTX-4 may be transported from chloroplasts to vacuoles at the periphery of the cells by light-mediated transporters. In vacuoles, DTX-4 convert to the OA-diol ester, which transform into either OA or DTX 1. The dot line in the figure indicated as the postulated parts of the pathway.

After consolidating different findings from the literature, we postulated a possible pathway of DSP-toxin production regarding hybrid NRPS/PKS (Figure 2). Because the expression of partial transcripts—*mcyB* and *mcyD*—in cyanobacterial NRPS/PKS is regulated by light, the pathway may trigger the transcription and translation of *NRPS/PKS* [143]. Glycolate and acetate from photorespiration and glyoxylate cycle, respectively, may act as raw materials of DSP toxin biosynthesis. According to suggestion in Section 2.2, remained glycerol from uptake of glycerophorphate may provide additional carbon source to enhance the cellular toxicity though increase the yield of glycolate and acetate. In chloroplasts, DTX-5b is produced by hybrid NRPS/PKS, and it may transform into DTX-5a through single carbon deletion. The amide group of glycine in DTX-5a may be removed by NRPS/PKS and become DTX-4. As neither DTX-5 nor DTX4 inhibit PP2A activity, these toxin derivatives can be synthesised in the chloroplast. Water-soluble DTX-4 in chloroplasts may then be transported to the vacuoles at the periphery of cells by a potential light-mediated transporter to prevent the DSP toxin-mediated inhibition of PP2A inside the cells [61,89]. When the cell cycle changes from the G1 to G2 phase, DTX-4 may be enzymatically converted into the OA-diol ester, which would transform into either OA or DTX-1 [61]. This postulated pathway is still working even though the cell is under low nitrogen amount, low phosphorus amount and extreme temperatures with unfavourable growth. Furthermore, higher cellular toxin level with unfavourable growth implicates the accumulation of toxin due to non-stop working of the pathway.

## 7. Conclusions and Perspectives

**Figure 3:**
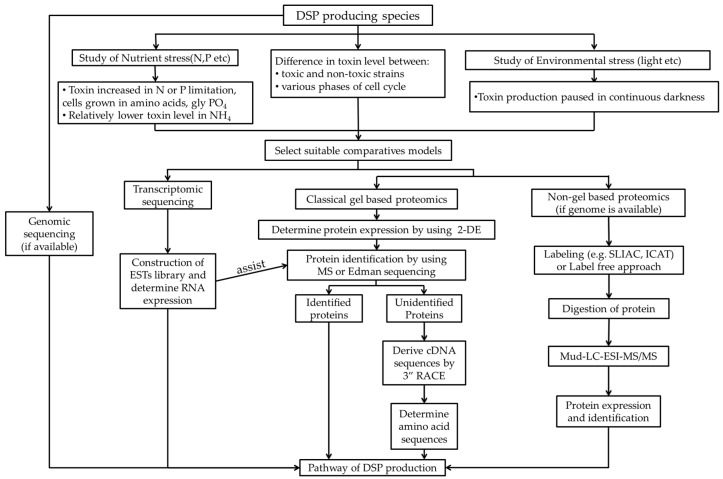
A flowchart summarising the potential molecular studies of the DSP toxin production mechanism. Abbreviation: N, nitrogen; P, phosphorus; Gly PO_4_, glycerophosphate; NH_4_, ammonium; ESTs, expressed sequence tags; SLIAC, stable isotope labelling by amino acid; ICAT, isotope-coded affinity tag; 2-DE, two-dimensional gel electrophoresis; RACE, rapid amplification of cDNA ends.

Alterations in nutritional and environmental factors, such as nitrogen or phosphorus limitation and low salinity, may vary the levels of DSP toxins in *Prorocentrum* spp. Molecular investigations into toxin production under various nutritional and environmental factor alterations can facilitate the study of the association between different conditions and the DSP toxin production pathway (Figure 3). Under the stimulation of various factors, time points of interest, such as those showing considerable variations in cellular toxicity, can be selected for comparative omics analysis. RNA identity and expression can be determined through transcriptomics analyses, whereas protein expression can be studied through gel-based proteomics analyses. If proteins cannot be identified, de novo peptide sequences can be derived using LC–MS/MS. The amino acid sequences of the target protein can then be deduced from the corresponding cDNA sequences. If a genome is available, non-gel-based proteomics can be conducted for protein identification; however, the genome data of dinoflagellates are incomplete to date. Finally, with the assistance from the complete genome data of *Symbiodinium* spp. and the development of advanced NGS technologies, these difficult topics may become feasible for study in the near future [93].

## Figures and Tables

**Table 1:** The growth and toxin levels of *Prorocentrum lima* (*P. lima*) under different concentrations and sources of nitrogen.

Strains	N Source: P Source	Initial N (μM):P (μM)	N/P		Growth Rate (μ, Day^−1^)	Max. Cell Density (Cells/mL)	Cellular Toxin Level (pg/Cell)	Reference
*P. lima* (Isolates from coastal lagoon of Goro)	NO_3_:PO_4_	17.7:36.3	0.49	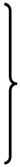	0.22	7040	OA: 12.5 ^b^, DTX-1:0.39 ^b^	[15]
		44.2:36.3	1.22			~10000 ^c^	OA: ~11 ^b,c^, DTX-1: ~0.25 ^b,c^	
		88.3:36.3	2.43			~14000 ^c^	OA: ~11.74 ^b,c^, DTX-1: ~0.18 ^b,c^	
		294:36.3	8.10			~24000 ^c^	OA: ~10.34 ^b,c^, DTX-1: ~ 0.15 ^b,c^	
		882:36.3	24.30		0.23	33100–35000	OA: 6.69–6.87 ^b^, DTX-1: 0.12 ^b^	
*P. lima* (CCAP1136/11)	NO_3_:PO_4_	882:36.3	24.30		0.22	20950	OA: ~6 ^b,c^	[16]
	NH_4_:PO_4_	882:36.3	24.30		0.21	10790	OA:~4.7 ^b,c^	
*P. lima* (CCMP2579)	NO_3_:PO_4_	88.2:36.3	2.43		~0.029 ^a^	~5220 ^c^	OA: 192.69 ^b^	[12]
		882:36.3	24.30		~0.039 ^a^	~10000 ^c^	OA: 100.66 ^b^	
*P. lima* (CCMP2579)	NO_3_:PO_4_	12:36.3	0.33	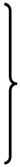	0.058	~13180 ^c^	OA: ~210 ^b,c^	[14]
		25:36.3	0.69			~13180 ^c^	OA: ~275 ^b,c^	
		50:36.3	1.38			~14540 ^c^	OA: ~240 ^b,c^	
		100:36.3	2.75			~15000 ^c^	OA: ~338 ^b,c^	
	NH_4_:PO_4_	12:36.3	0.33	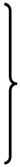	0.059	~13210 ^c^	OA: ~85 ^b,c^	
		25:36.3	0.69			~11740 ^c^	OA: ~68 ^b,c^	
		50:36.3	1.38			~12500 ^c^	OA: ~85 ^b,c^	
		100:36.3	2.75			~13620 ^c^	OA: ~100 ^b,c^	
	Urea:PO_4_	12:36.3	0.33	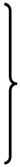	0.060	~13880 ^c^	OA: ~100 ^b,c^	
		25:36.3	0.69			~13330 ^c^	OA: ~58 ^b,c^	
		50:36.3	1.38			~15550 ^c^	OA: ~55 ^b,c^	
		100:36.3	2.75			18330	OA: ~113 ^b,c^	
*P. lima* (Clone from Mahone Bay, Nova Scotia)	NO_3_:PO_4_	0:36.3	0.00		~0.013 ^a^	~8077 ^c^	OA+DTX-1 at Day 20: 34 ^b^	[17]
		300:36.3	8.26		~0.032 ^a^	~20000 ^c^	OA+DTX-1 at Day 30: 25 ^b^	
		1000:36.3	27.55		~0.033 ^a^	~20833 ^c^	OA+DTX-1 at Day 30: 7 ^b^	
*P. lima* (CCMP2579)	NO_3_:PO_4_	17.7:36.3	0.49		~0.103 ^a^	~35454 ^c^	OA: ~30.1 ^b,c^	[23]
		882:36.3	24.30		~0.097 ^a^	~40000 ^c^	OA: ~17.5 ^b,c^	

^a^ The specific growth rate (μ) is calculated according to the formula shown below; $\mu =\frac{\ln N_{1}-\ln N_{0}}{t_{1}-t_{0}}$ where *N*_0_ and *N*_1_ are the cell density reading at time *t*_0_ and *t*_1_; ^b^ The toxin level was determined at stationary phase; ^c^ The data were estimated from data in the corresponding publication; ^d^ NO_3_, nitrate; NH_4_, ammonium; PO_4_, phosphate; OA, okadaic acid; DTX-1, dinophysistoxin-1.

**Table 2:** The growth and toxin levels of *Prorocentrum* under different concentrations and sources of phosphorus.

Strains	N Source:P Source	Initial N(μM):P(μM)	N/P		Growth Rate (μ, day^−1^)	Max. Cell Density (cells/mL)	Cellular Toxin Level (pg/cell)	Reference
*P. lima* (Isolates from coastal lagoon of Goro)	NO_3_:PO_4_	882:0.73	1208.22	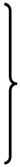	0.22	6250	OA: 10.75 ^b^, DTX-1: ~ 0.26 ^b,c^	[15]
		882:1.81	487.29			~11,000 ^c^	OA: 15.80 ^b^, DTX-1: 0.32 ^b^	
		882:3.63	242.98			~17,000 ^c^	OA: ~11.27 ^b,c^, DTX-1: ~ 0.26 ^b,c^	
		882:12.1	72.89			~31,000 ^c^	OA: ~9.7 ^b,c^, DTX-1: ~ 0.16 ^b,c^	
		882:36.3	24.30		0.23	33,100–35,000	OA: 6.69–6.87 ^b^, DTX-1: 0.13 ^b^	
*P. lima* (CCMP2579)	NO_3_:PO_4_	882:3.63	242.98		~0.029 ^a^	~3333 ^c^	OA: 268.68 ^b^	[12]
		882:36.3	24.30		~0.039 ^a^	~10,000 ^c^	OA: 100.66 ^b^	
*P. lima* (CCMP2579)	NO_3_:PO_4_	882:0.5	1764.00	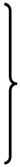	0.059	~12,230 ^c^	OA: ~88 ^b c^	[13]
		882:1	882.00			~13,290 ^c^	OA: ~76 ^b,c^	
		882:2	441.00			~14,200 ^c^	OA: ~86 ^b,c^	
		882:5	176.40			~15,000 ^c^	OA: ~60 ^b,c^	
		882:10	88.20			~16,000 ^c^	OA: ~68 ^b,c^	
	NO_3_:Gly PO_4_	882: 0.5	1764.00	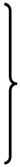	0.048	~11,840 ^c^	OA: ~116 ^b,c^	
		882:1	882.00			~11,840 ^c^	OA: ~97 ^b,c^	
		882:2	441.00			~11,440 ^c^	OA: ~100 ^b,c^	
		882:5	176.40			~13,600 ^c^	OA: ~94 ^b,c^	
		882:10	88.20			~16,310 ^c^	OA: ~88 ^b,c^	
	NO_3_:ATP	882:0.5	1764.00	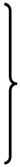	0.053	~13,160 ^c^	OA:~51 ^b,c^	
		882:1	882.00			~13,830 ^c^	OA: ~57 ^b,c^	
		882:2	441.00			~17,000^c^	OA: ~57 ^b,c^	
		882:5	176.40			~18,160 ^c^	OA: ~60 ^b,c^	
		882:10	88.20			~19,160 ^c^	OA: ~57 ^b,c^	
*P. lima* (Isolates from dry Tortugas, Florida)	NO_3_ + NH_4_:PO_4_	932:10	93.20		~0.5 ^a^	135,185	OA at Day30: 8.9 ^b^	[18]
	NO_3_ + NH_4_:gly PO_4_	932:10	93.20		~0.144 ^a^	221,445	OA at Day30: 14.2 ^b^	
*P. lima* (CCMP685)	NO_3_:Gly PO_4_	882:10	88.20		~0.082 ^a^	26,637 at Day 40	OA at Day47: 9.96	[19]
		882:20	44.10		~0.094 ^a^	43,128 at Day 40	N/A	
		882: 30	29.40		~0.101 ^a^	55,903 at Day 40	OA at Day47: ~4.4 ^c^	
	NO_3_:PO_4_	882:10	88.20		~0.08 ^a^	25,014 at Day 40	OA at Day47: ~7 ^c^	
		882:20	44.10		~0.092 ^a^	39,997 at Day 40	N/A	
		882:30	29.40		~0.098 ^a^	50,016 at Day 40	OA at Day47: ~4 ^c^	
*P. lima* (CCMP2579)	NO_3_:PO_4_	882:1.81	487.29		~0.021 ^a^	~25,909 ^c^	OA: ~35.2 ^b,c^	[23]
		882:36.3	24.30		~0.097 ^a^	~40,000 ^c^	OA: ~17.5 ^b,c^	

^a^ The specific growth rate is calculated according to the formula shown below; $\mu =\frac{\ln N_{1}-\ln N_{0}}{t_{1}-t_{0}}$ where *N*_0_ and *N*_1_ are the cell density reading at time *t*_0_ and *t*_1_; ^b^ The toxin level was determined at stationary phase; ^c^ The data were estimated from data in the corresponding publication; ^d^ NO_3_, nitrate; NH_4,_ ammonium; PO_4_, phosphate; Gly PO_4_, glycerophospahte; ATP, adenosine triphosphate; OA, okadaic acid; DTX-1, dinophysistoxin-1.

**Table 3:** The growth and toxin levels of *Prorocentrum lima*, *P. concavum*, *P. hoffmannianum*, and *P. belizeanum* under different temperatures.

Strains	Temperature (°C)	Growth Rate (μ, day^−1^)	Max. Cell Density (Cells/mL)	Cellular Toxin Level (pg/cell)	Reference
*P. lima* (Isolates from Nova Scotia, Canada)	5 °C	~0.000 ^a^	~1000 at Day 28	OA at Day 28:8	[20]
	10 °C	~0.073 ^a^	~7800 at Day 28	OA at Day 28:2.5	
	15 °C	~0.150 ^a^	~11,000 at Day 16	OA at Day 16:4.4	
	20 °C	~0.198 ^a^	~23,800 at Day 16	OA at Day 16:2.5	
	25 °C	~0.229 ^a^	~39,000 at Day 16	OA at Day 16:1.4	
*P. lima* (PL100A, Isolates from Knight Key, Florida)	19–33 °C	~0.05 ^c^–0.3 (max. at 26 °C)	N/A	N/A	[59]
*P. lima* (PL100A)	21–33 °C	~0.066–~0.3 ^a^ (max. at 27 °C)	N/A	N/A	[60]
*P. concavum* (PC100A, Isolates from Knight Key, Florida)	21–31 °C	~0.05–~0.28 ^a^ (max. at 26 °C)	N/A	N/A	
*P. hoffmannianum* (882a, Isolates from Little Lameshur Bay, St. John, US Virgin Island)	23–31 °C	- 2000 lux: ~0.06–~0.2 ^c^ (max. at 27 °C) - 3000 lux: ~0.1–~0.425 ^c^ (max. at 29 °C) - 4000 lux: ~0.07–~0.425 ^c^ (max. at 27 °C) - 5000 lux: ~0.08–~0.53 ^c^ (max. at 27 °C)	N/A	- OA at 2000 lux:~10–~53.75 ^c^ (max. at 23 °C) - OA at 3000 lux:~11.25–~38 ^c^ (max. at 29 °C) - OA at 4000 lux:~5–45 ^c^ (max. at 29 °C) - OA at 5000 lux: ~2.5 ~ 17.5 ^c^ (max. at 25 and 31 °C)	[21]
*P. belizeanum* (VGO1029, Isolates from La Puntilla, Las Palmas de Gran Canaria)	18 °C	~0.08–0.125 ^a^ (max. at 40 µE m^−2^ s^−1^)	~135,000 ^c^ at 40 µE m^−2^ s^−1^	OA: ~4.75 ^b,c^	[24]
	25 °C	~0.06–0.205 ^a^ (max. at 40 µE m^−2^ s^−1^)	~120,000 ^c^ at 40 µE m^−2^ s^−1^	OA: ~1.1 ^b,c^	
	28 °C	−0.075–0.125 ^a^ (max. at 40 µE m^−2^ s^−1^)	~40,000 ^c^ at 40 µE m^−2^ s^−1^	OA: ~2.625 ^b,c^	
*P. lima* (CCMP2579)	15 °C	~0.048 ^a^	~16,700 ^c^	OA:~5.5 ^b,c^, DTX-1: ~14.1 ^b,c^	[22]
	20 °C	~0.050 ^a^	~25,700 ^c^	OA:~1.5 ^b,c^, DTX-1: ~11 ^b,c^	
	25 °C	~0.056 ^a^	~25,100 ^c^	OA:~1 ^b,c^, DTX-1: ~4.7 ^b,c^	
	30 °C	~0.036 ^a^	~8200 ^c^	OA:~12.731 ^b^, DTX-1: ~16.587 ^b^	

^a^ The specific growth rate is calculated according to the formula shown below; $\mu =\frac{\ln N_{1}-\ln N_{0}}{t_{1}-t_{0}}$ where *N*_0_ and *N*_1_ are the cell density reading at time *t*_0_ and *t*_1_; ^b^ The toxin level was determined at stationary phase; ^c^ The data were estimated from data in the corresponding publication. d. OA, okadaic acid; DTX-1.

## References

[1] Kat M. (1983). Diarrhetic mussel poisoning in the Netherlands related to the dinoflagellate *Dinophysis acuminata*. Antonie van Leeuwenhoek.

[2] Aune T., Yndestad M., Ian R.F. (1993). Diarrhetic shellfish poisoning. Algal Toxins in Seafood and Drinking Water.

[3] Hallegraeff G.M. (1993). A review of harmful algal blooms and their apparent global increase. Phycologia.

[4] Pitcher G.C., Krock B., Cembella A.D. (2011). Accumulation of diarrhetic shellfish poisoning toxins in the oyster *Crassostrea gigas* and the mussel *Choromytilus meridionalis* in the Southern Benguela ecosystem. Afr. J. Mar. Sci..

[5] Li A., Ma J., Cao J., McCarron P. (2012). Toxins in mussels (*Mytilus galloprovincialis*) associated with diarrhetic shellfish poisoning episodes in China. Toxicon.

[6] Madigan T.L., Lee K.G., Padula D.J., McNabb P., Pointon A.M. (2006). Diarrhetic shellfish poisoning (DSP) toxins in South Australian shellfish. Harmful Algae.

[7] Sim J., Wilson N. (1997). Surveillance of marine biotoxins, 1993–96. N. Z. Public Health Rep..

[8] Fernández J.J., Suárez-Gómez B., Souto M.L., Norte M. (2003). Identification of new okadaic acid derivatives from laboratory cultures of *Prorocentrum lima*. J. Nat. Prod..

[9] Yasumoto T., Oshima Y., Sugawara W., Fukuyo Y., Oguri H., Igarashi T., Fujita N. (1980). Identification of *Dinophysis fortii* as the causative organism of diarrhetic shellfish poisoning. Nippon Suisan Gakk.

[10] Zhou J., Fritz L. (1994). Okadaic acid antibody localizes to chloroplasts in the DSP-toxin-producing dinoflagellates *Prorocentrum lima* and *Prorocentrum maculosum*. Phycologia.

[11] Granéli E., Flynn K., Granéli E., Turner J.T. (2006). Chemical and physical factors influencing toxin content. Ecology of Harmful Algae.

[12] Li L.X., Tang J.Q., Yang W.D., Liu J.S., Zhang J.J., Li H.Y. (2009). Studies on toxin production of *Prorocentrum lima*. Asian J. Ecotoxicol..

[13] Yang W.d., Zhong N., Liu J.S., Zhang J.L., Yang H. (2008). Effects of different phosphorus sources on the growth and toxin production of *Prorocentrum lima*. Environ. Sci..

[14] Zhong N., Yang W., Liu J., Zhang J., He Y. (2008). Effects of different nitrogen sources on the growth and toxin production of *Prorocentrum lima*. Acta Sci. Circumst..

[15] Vanucci S., Guerrini F., Milandri A., Pistocchi R. (2010). Effects of different levels of N- and P-deficiency on cell yield, okadaic acid, DTX-1, protein and carbohydrate dynamics in the benthic dinoflagellate *Prorocentrum lima*. Harmful Algae.

[16] Varkitzi I., Pagou K., Granéli E., Hatzianestis I., Pyrgaki C., Pavlidou A., Montesanto B., Economou-Amilli A. (2010). Unbalanced N:P ratios and nutrient stress controlling growth and toxin production of the harmful dinoflagellate *Prorocentrum lima* (Ehrenberg) dodge. Harmful Algae.

[17] McLachlan J.L., Marr J.C., Conlon-Keily A., Adamson A. (1994). Effects of nitrogen concentration and cold temperature on DSP-toxin concentrations in the dinoflagellate *Prorocentrum lima* (Prorocentrales, Dinophyceae). Nat. Toxins.

[18] Carmelo R.T., Daniel G.B., Smayda T.J., Shimizu Y. (1993). The influence of phosphorus sources on the growth and cellular toxin content of the benthic dinoflagellate *Prorocentrum lima*. Toxic Phytoplankton Blooms in the Sea.

[19] Sohet K., Pereira A., Braekman J.C., Houvenaghel G., Lassus P., Arzul G., Erard E., Gentlen P., Marcailiou C. (1995). Growth and toxicity of *Prorocentrum lima* (Ehrenberg) dodge in different culture media: Effect of humic acids and organic phosphorus. Harmful Marine Algal Blooms: Proceedings of the Sixth International Conference on Toxic Marine Phytoplankton.

[20] Jackson A.E., Marr J.C., McLachlan J.L., Smayda T.J., Shimizu Y. (1993). The production of diarrhetic shellfish toxins by an isolate of *Prorocentrum lima* from Nova Soctia, Canada. Toxic Phytoplankton Blooms in the Sea: Proceedings of the Fifth International Conference on Toxic Marine Phytoplankton.

[21] Morton S.L., Bomber J.W., Tindall P.M. (1994). Environmental effects on the production of okadaic acid from *Prorocentrum*
*hoffmannianum*
*faust* I. Temperature, light, and salinity. J. Exp. Mar. Biol. Ecol..

[22] Wang S., Chen J., Li Z., Wang Y., Fu B., Han X., Zheng L. (2015). Cultivation of the benthic microalga *Prorocentrum lima* for the production of diarrhetic shellfish poisoning toxins in a vertical flat photobioreactor. Bioresour. Technol..

[23] Hou D.Y., Liang J.J., Zou C., Li H.Y., Liu J.S., Yang W.D. (2016). MRP functional activity and character in the dinoflagellate *Prorocentrum lima*. J. Appl. Phycol..

[24] López-Rosales L., Gallardo-Rodríguez J., Sánchez-Mirón A., Cerón-García M., Belarbi E., García-Camacho F., Molina-Grima E. (2014). Simultaneous effect of temperature and irradiance on growth and okadaic acid production from the marine dinoflagellate *Prorocentrum belizeanum*. Toxins.

[25] Blanco J., Moroño Á., Fernández M.L. (2005). Toxic episodes in shellfish, produced by lipophilic phycotoxins: An overview. Revista Galega de Recursos Mariños (Monog.).

[26] Cohen P., Holmes C.F.B., Tsukitani Y. (1990). Okadaic acid: A new probe for the study of cellular regulation. Trends Biochem. Sci..

[27] EFSA Panel on Contaminants in the Food Chain (CONTAM) (2008). Opinion of the scientific panel on contaminants in the food chain on a request from the european commission on marine biotoxins in shellfish – okadaic acid and analogues. EFSA J..

[28] Tachibana K., Scheuer P.J., Tsukitani Y., Kikuchi H., Van Engen D., Clardy J., Gopichand Y., Schmitz F.J. (1981). Okadaic acid, a cytotoxic polyether from two marine sponges of the genus *Halichondria*. J. Am. Chem. Soc..

[29] Schmitz F.J., Prasad R.S., Gopichand Y., Hossain M.B., Van der Helm D., Schmidt P. (1981). Acanthifolicin, a new episulfide-containing polyether carboxylic acid from extracts of the marine sponge *Pandaros acanthifolium*. J. Am. Chem. Soc..

[30] Dominguez H.J., Paz B., Daranas A.H., Norte M., Franco J.M., Fernández J.J. (2010). Dinoflagellate polyether within the yessotoxin, pectenotoxin and okadaic acid toxin groups: Characterization, analysis and human health implications. Toxicon.

[31] Yasumoto T., Murata M., Oshima Y., Sano M., Matsumoto G.K., Clardy J. (1985). Diarrhetic shellfish toxins. Tetrahedron.

[32] García C., Truan D., Lagos M., Santelices J.P., Dêaz J.C., Lagos N. (2005). Metabolic transformation of dinophysistoxin-3 into dinophysistoxin-1 causes human intoxication by consumption of O-acyl-derivatives dinophysistoxins contaminated shellfish. J. Toxicol. Sci..

[33] Suzuki T., Kamiyama T., Okumura Y., Ishihara K., Matsushima R., Kaneniwa M. (2009). Liquid-chromatographic hybrid triple–quadrupole linear-ion-trap ms/ms analysis of fatty-acid esters of dinophysistoxin-1 in bivalves and toxic dinoflagellates in japan. Fisheries Sci..

[34] Vanden Heuvel J.P. (2002). Xenobiotic receptor systems: Introduction and overview. Comprehensive Toxicology: Cellular and Molecular Toxicology.

[35] Suganuma M., Fujiki H., Suguri H., Yoshizawa S., Hirota M., Nakayasu M., Ojika M., Wakamatsu K., Yamada K., Sugimura T. (1988). Okadaic acid: An additional non-phorbol-12-tetradecanoate-13-acetate-type tumor promoter. Proc. Natl. Acad. Sci. USA.

[36] Fujiki H., Suganuma M., Suguri H., Yoshizawa S., Takagi K., Uda N., Wakamatsu K., Yamada K., Murata M., Yasumoto T. (1988). Diarrhetic shellfish toxin, dinophysistoxin-1, is a potent tumor promoter on mouse skin. Cancer Sci..

[37] Fujiki H., Suganuma M. (1993). Tumor promotion by inhibitors of protein phosphatases 1 and 2a: The okadaic acid class of compounds. Adv. Cancer Res..

[38] Cordier S., Monfort C., Miossec L., Richardson S., Belin C. (2000). Ecological analysis of digestive cancer mortality related to contamination by diarrhetic shellfish poisoning toxins along the coasts of france. Environ. Res..

[39] Bøe R., Gjertsen B.T., Vintermyr O.K., Houge G., Lanotte M., Døskeland S.O. (1991). The protein phosphatase inhibitor okadaic acid induces morphological changes typical of apoptosis in mammalian cells. Exp. Cell Res..

[40] Lerga A., Richard C., Delgado M.D., Cañelles M., Frade P., Cuadrado M.A., León J. (1999). Apoptosis and mitotic arrest are two independent effects of the protein phosphatases inhibitor okadaic acid in K562 leukemia cells. Biochem. Bioph. Res. Commun..

[41] Jayaraj R., Gupta N., Rao P.V.L. (2009). Multiple signal transduction pathways in okadaic acid induced apoptosis in hela cells. Toxicology.

[42] Traoré A., Baudrimont I., Ambaliou S., Dano S.D., Creppy E.E. (2001). DNA breaks and cell cycle arrest induced by okadaic acid in Caco-2 cells, a human colonic epithelial cell line. Arch. Toxicol..

[43] Chen L. (2011). Okadaic acid induces apoptosis through the PKR, NF-κB and caspase pathway in human osteoblastic osteosarcoma MG63 cells. Toxicol. in Vitro.

[44] Nuydens R., De Jong M., Van Den Kieboom G., Heers C., Dispersyn G., Cornelissen F., Nuyens R., Borgers M., Geerts H. (1998). Okadaic acid-induced apoptosis in neuronal cells: Evidence for an abortive mitotic attempt. J. Neurochem..

[45] Souid-Mensi G., Moukha S., Mobio T.A., Maaroufi K., Creppy E.E. (2008). The cytotoxicity and genotoxicity of okadaic acid are cell-line dependent. Toxicon.

[46] Berven G., Sætre F., Halvorsen K., Seglen P.O. (2001). Effects of the diarrhetic shellfish toxin, okadaic acid, on cytoskeletal elements, viability and functionality of rat liver and intestinal cells. Toxicon.

[47] Fessard V., Grosse Y., Pfohl-Leszkowicz A., Puiseux-Dao S. (1996). Okadaic acid treatment induces DNA adduct formation in BHK21 C13 fibroblasts and HESV keratinocytes. Mutat. Res..

[48] Hégarat L.L., Orsière T., Botta A., Fessard V. (2005). Okadaic acid: Chromosomal non-disjunction analysis in human lymphocytes and study of aneugenic pathway in CHO-K1 cells. Mutat. Res..

[49] Louzao M.C., Fernández D.A., Abal P., Fraga M., Vilariño N., Vieytes M.R., Botana L.M. (2015). Diarrhetic effect of okadaic acid could be related with its neuronal action: Changes in neuropeptide y. Toxicol. Lett..

[50] Bechemin C., Grzebyk D., Hachame F., Hummert C., Maestrini S.Y. (1999). Effect of different nitrogen/phosphorus nutrient ratios on the toxin content in *Alexandrium minutum*. Aquat. Microb. Ecol..

[51] Flynn K., Franco J.M., Fernandez P., Reguera B., Zapata M., Wood G., Flynn K.J. (1994). Changes in toxin content, biomass and pigments of the dinoflagellate *Alexandrium*
*minutum* during nitrogen refeeding and growth into nitrogen or phosphorus stress. Mar. Ecol. Prog. Ser..

[52] Hwang D.F., Lu Y.H. (2000). Influence of environmental and nutritional factors on growth, toxicity, and toxin profile of dinoflagellate *Alexandrium minutum*. Toxicon.

[53] John E.H., Flynn K.J. (2000). Growth dynamics and toxicity of *Alexandrium fundyense* (Dinophyceae): The effect of changing n:P supply ratios on internal toxin and nutrient levels. Eur. J. Phycol..

[54] Oh S.J., Yamamoto T., Kataoka Y., Matsuda O., Matsuyama Y., Kotani Y. (2002). Utilization of dissolved organic phosphorus by the two toxic dinoflagellates, *Alexandrium tamarense* and *Gymnodinium catenatum* (dinophyceae). Fish. Sci..

[55] Wang D.Z., Hsieh D.P.H. (2002). Effects of nitrate and phosphate on growth and C2 toxin productivity of *Alexandrium tamarense* CI01 in culture. Mar. Pollut. Bull..

[56] Murata A., Leong S.C.Y., Nagashima Y., Taguchi S. (2006). Nitrogen:Phosphorus supply ratio may control the protein and total toxin of dinoflagellate *Alexandrium tamarense*. Toxicon.

[57] Lee T.C.H., Kwok O.T., Ho K.C., Lee F.W.F. (2012). Effects of different nitrate and phosphate concentrations on the growth and toxin production of an *Alexandrium tamarense* strain collected from Drake passage. Mar. Environ. Res..

[58] Xu J., Ho A.Y.T., He L., Yin K., Hung C., Choi N., Lam P.K.S., Wu R.S.S., Anderson D.M., Harrison P.J. (2012). Effects of inorganic and organic nitrogen and phosphorus on the growth and toxicity of two *Alexandrium* species from hong kong. Harmful Algae.

[59] Morton S.L., Norris D.R., Granéli E., Sundstorm B., Edler L., Anderson D.M. (1990). Role of temperature, salinity, and light on the seasonality of *Prorocentrum lima* (ehrenberg) dodge. Toxic Marine Phytoplankton: Proceedings of the Fourth International Conference on Toxic Marine Phytoplankton.

[60] Morton S.L., Norris D.R., Bomber J.W. (1992). Effect of temperature, salinity and light intensity on the growth and seasonality of toxic dinoflagellates associated with ciguatera. J. Exp. Mar. Biol. Ecol..

[61] Pan Y., Cembella A.D., Quilliam M.A. (1999). Cell cycle and toxin production in the benthic dinoflagellate *Prorocentrum lima*. Mar. Biol..

[62] Park M.G., Kim S., Kim H.S., Myung G., Kang Y.G., Yih W. (2006). First successful culture of the marine dinoflagellate *Dinophysis acuminata*. Aquat. Microb. Ecol..

[63] Nielsen L.T., Krock B., Hansen P.J. (2013). Production and excretion of okadaic acid, pectenotoxin-2 and a novel dinophysistoxin from the DSP-causing marine dinoflagellate *Dinophysis acuta*—Effects of light, food availability and growth phase. Harmful Algae.

[64] Tong M., Kulis D.M., Fux E., Smith J.L., Hess P., Zhou Q., Anderson D.M. (2011). The effects of growth phase and light intensity on toxin production by *Dinophysis acuminata* from the Northeastern United States. Harmful Algae.

[65] Smith J.L., Tong M., Fux E., Anderson D.M. (2012). Toxin production, retention, and extracellular release by *Dinophysis acuminata* during extended stationary phase and culture decline. Harmful Algae.

[66] Lourenço S.O., Barbarino E., Lavín P.L., Lanfer Marquez U.M., Aidar E. (2004). Distribution of intracellular nitrogen in marine microalgae: Calculation of new nitrogen-to-protein conversion factors. Eur. J. Phycol..

[67] Lai J., Yu Z., Song X., Cao X., Han X. (2011). Responses of the growth and biochemical composition of *Prorocentrum donghaiense* to different nitrogen and phosphorus concentrations. J. Exp. Mar. Biol. Ecol..

[68] Cary L.B., Jonathan R.P. (2000). The effect of nitrogen source on the growth and toxicity of species of the genus *Prorocentrum*. Symposium on Harmful Marine Algae in the U.S..

[69] Souto M.L., Fernández J.J., Norte M., Fernández M.L., Martínez A. (2001). Influence of amino acids on okadaic acid production. Toxicon.

[70] Aikman K.E., Tindall D.R., Morton S.L., Smayda T.J., Shimizu Y. (1993). Physiology, potency of the dinoflagellate *Prorocentrum hoffmannianum* (faust) during one complete growth cycle. Toxic Phytoplankton Blooms in the Sea.

[71] Morel F.M.M., Milligan A.J., Saito M.A., Elderfield H., Holland H.D., Turekian K.K. (2006). Marine bioinorganic chemistry: The role of trace metals in the oceanic cycles of major nutrients. The Oceans and Marine Geochemistry.

[72] Azad H.S., Borchardt J.A. (1970). Variations in phosphorus uptake by algae. Environ. Sci. Technol..

[73] Nascimento S.M., Purdie D.A., Morris S. (2005). Morphology, toxin composition and pigment content of *Prorocentrum lima* strains isolated from a coastal lagoon in Southern UK. Toxicon.

[74] Glibert P.M., Legrand C., Granéli E., Turner J.T. (2006). The diverse nutrient strategies of harmful algae: Focus on osmotrophy. Ecology of Harmful Algae.

[75] Andersen R.A. (2005). Algal Culturing Techniques.

[76] Tindall D.R., Morton S.L., Anderson D.M., Cembella A.D., Hallegraeff G.M. (1998). Community dynamics and physiology of epiphytic/benthic dinoflagellates associated with ciguatera. Physiological Ecology of Harmful Algal Blooms.

[77] Faust M.A. (1993). Three new benthic species of *Prorocentrum* (dinophyceae) from Twin Cays, Belize: *P.*
*maculosum* sp. nov., *P.*
*foraminosum* sp. nov. and *P.*
*formosum* sp. nov.. Phycologia.

[78] Aligizaki K., Nikolaidis G., Katikou P., Baxevanis A.D., Abatzopoulos T.J. (2009). Potentially toxic epiphytic *Prorocentrum* (Dinophyceae) species in greek coastal waters. Harmful Algae.

[79] Glibert P.M., Burkholder J.M., Kana T.M. (2012). Recent insights about relationships between nutrient availability, forms, and stoichiometry, and the distribution, ecophysiology, and food web effects of pelagic and benthic *Prorocentrum* species. Harmful Algae.

[80] Koike K., Sato S., Yamaji M., Nagahama Y., Kotaki Y., Ogata T., Kodama M. (1998). Occurrence of okadaic acid-producing *Prorocentrum lima* on the sanriku coast, northern japan. Toxicon.

[81] Samuelsson G., Richardson K. (1982). Photoinhibition at low quantum flux densities in a marine dinoflagellate (*Amphidinium carterae*). Mar. Biol..

[82] Woodward F.I., Sheehy J.E., Woodward F.I., Sheehy J.E. (1983). Radiation. Principles and Measurements in Environmental Biology.

[83] Kiang N.Y., Siefert J., Blankenship R.E. (2007). Spectral signatures of photosynthesis. I. Review of earth organisms. Astrobiology.

[84] Tanaka K., Iida S., Takaichi S., Mimuro M., Murakami A., Akimoto S. (2016). Excitation relaxation dynamics and energy transfer in pigment–protein complexes of a dinoflagellate, revealed by ultrafast fluorescence spectroscopy. Photosynth. Res..

[85] Rausch de Traubenberg C., Géraud M.L., Soyer-Gobillard M.O., Emdadi D. (1995). The toxic dinoflagellate *Prorocentrum lima* and its associated bacteria: I. An ultrastructural study. Eur. J. Protistol..

[86] Prokic I., Brümmer F., Brigge T., Görtz H.D., Gerdts G., Schütt C., Elbrächter M., Müller W.E.G. (1998). Bacteria of the genus *Roseobacter* associated with the toxic dinoflagellate *Prorocentrum lima*. Protist.

[87] Lafay B., Ruimy R., Rausch De Traubenber C., Breittmayer V., Gauthier M.J., Christen R. (1995). *Roseobacter algicola* sp. Nov., a new marine bacterium isolated from the phycosphere of the toxin-producing dinoflagellate *Prorocentrum lima*. Int. J. Syst. Bacteriol..

[88] Rausch de Traubenberg C. (1993). Interactions between a Dinoflagellate and It Associated Bacterial Microflora: Role of Bacteria in the Toxicity of *Prorocentrun lima* Ehrenberg (Dodge). Ph.D. Thesis.

[89] Barbier M., Amzil Z., Mondeguer F., Bhaud Y., Soyer-Gobillard M.-O., Lassus P. (1999). Okadaic acid and PP2A cellular immunolocalization in *Prorocentrum lima* (dinophyceae). Phycologia.

[90] Spector D.L., Spector D.L. (1984). Dinoflagellate nuclei. Dinoflagellates.

[91] Rizzo P.J., Taylor F.J.R. (1987). Biochemistry of the dinoflagellate nucleus. The Biology of Dinoflagellates.

[92] Hackett J.D., Anderson D.M., Erdner D.L., Bhattacharya D. (2004). Dinoflagellates: A remarkable evolutionary experiment. Am. J. Bot..

[93] McLean T.I. (2013). “Eco-omics”: A review of the application of genomics, transcriptomics, and proteomics for the study of the ecology of harmful algae. Microb. Ecol..

[94] Yang I., John U., Beszteri S., Glockner G., Krock B., Goesmann A., Cembella A. (2010). Comparative gene expression in toxic versus non-toxic strains of the marine dinoflagellate *Alexandrium minutum*. BMC Genom..

[95] Yang I., Beszteri S., Tillmann U., Cembella A., John U. (2011). Growth- and nutrient-dependent gene expression in the toxigenic marine dinoflagellate *Alexandrium minutum*. Harmful Algae.

[96] Yang I., Beszteri S., Tillmann U., Cembella A., John U., Ho K.C., Zhou M.J., Qi Y.Z. (2008). Physiollogical and gene expression response to salinity stress in *Alexandrium minutum*. Proceedings of the 13th International Conference on Harmful Algae.

[97] Morey J., Monroe E., Kinney A., Beal M., Johnson J., Hitchcock G., Van Dolah F. (2011). Transcriptomic response of the red tide dinoflagellate, *Karenia brevis*, to nitrogen and phosphorus depletion and addition. BMC Genom..

[98] Bayer T., Aranda M., Sunagawa S., Yum L.K., DeSalvo M.K., Lindquist E., Coffroth M.A., Voolstra C.R., Medina M. (2012). *Symbiodinium* transcriptomes: Genome insights into the dinoflagellate symbionts of reef-building corals. PLoS ONE.

[99] Lowe C., Mello L., Samatar N., Martin L., Montagnes D., Watts P. (2011). The transcriptome of the novel dinoflagellate *Oxyrrhis marina* (Alveolata: Dinophyceae): Response to salinity examined by 454 sequencing. BMC Genom..

[100] Jaeckisch N., Yang I., Wohlrab S., Glöckner G., Kroymann J., Vogel H., Cembella A., John U. (2011). Comparative genomic and transcriptomic characterization of the toxigenic marine dinoflagellate *Alexandrium ostenfeldii*. PLoS ONE.

[101] Toulza E., Shin M.S., Blanc G., Audic S., Laabir M., Collos Y., Claverie J.-M., Grzebyk D. (2010). Gene expression in proliferating cells of the dinoflagellate *Alexandrium catenella* (dinophyceae). Appl. Environ. Microb..

[102] Wisecaver J., Hackett J. (2010). Transcriptome analysis reveals nuclear-encoded proteins for the maintenance of temporary plastids in the dinoflagellate *Dinophysis acuminata*. BMC Genom..

[103] Hackett J., Scheetz T., Yoon H., Soares M., Bonaldo M., Casavant T., Bhattacharya D. (2005). Insights into a dinoflagellate genome through expressed sequence tag analysis. BMC Genom..

[104] Tanikawa N., Akimoto H., Ogoh K., Chun W., Ohmiya Y. (2004). Expressed sequence tag analysis of the dinoflagellate *Lingulodinium polyedrum* during dark phase. Photochem. Photobiol..

[105] Lidie K.B., Ryan J.C., Barbier M., Dolah F.M. (2005). Gene expression in florida red tide dinoflagellate *Karenia brevis*: Analysis of an expressed sequence tag library and development of DNA microarray. Mar. Biotechnol..

[106] Velculescu V.E., Zhang L., Vogelstein B., Kinzler K.W. (1995). Serial analysis of gene expression. Science.

[107] Brenner S., Johnson M., Bridgham J., Golda G., Lloyd D.H., Johnson D., Luo S., McCurdy S., Foy M., Ewan M. (2000). Gene expression analysis by massively parallel signature sequencing (MPSS) on microbead arrays. Nat. Biotechnol..

[108] Dyhrman S.T. (2008). Molecular approaches to diagnosing nutritional physiology in harmful algae: Implications for studying the effects of eutrophication. Harmful Algae.

[109] Coyne K.J., Burkholder J.M., Feldman R.A., Hutchins D.A., Cary S.C. (2004). Modified serial analysis of gene expression method for construction of gene expression profiles of microbial eukaryotic species. Appl. Environ. Microb..

[110] Moustafa A., Evans A.N., Kulis D.M., Hackett J.D., Erdner D.L., Anderson D.M., Bhattacharya D. (2010). Transcriptome profiling of a toxic dinoflagellate reveals a gene-rich protist and a potential impact on gene expression due to bacterial presence. PLoS ONE.

[111] Erdner D., Anderson D. (2006). Global transcriptional profiling of the toxic dinoflagellate *Alexandrium fundyense* using massively parallel signature sequencing. BMC Genom..

[112] Zhang S., Sui Z., Chang L., Kang K., Ma J., Kong F., Zhou W., Wang J., Guo L., Geng H. (2014). Transcriptome de novo assembly sequencing and analysis of the toxic dinoflagellate *Alexandrium catenella* using the illumina platform. Gene.

[113] Zhang Y., Zhang S.F., Lin L., Wang D.Z. (2014). Comparative transcriptome analysis of a toxin-producing dinoflagellate *Alexandrium catenella* and its non-toxic mutant. Mar. Drugs.

[114] Okamoto O.K., Robertson D.L., Fagan T.F., Hastings J.W., Colepicolo P. (2001). Different regulatory mechanisms modulate the expression of a dinoflagellate iron-superoxide dismutase. J. Biol. Chem..

[115] Fagan T., Morse D., Hastings J.W. (1999). Circadian synthesis of a nuclear-encoded chloroplast glyceraldehyde-3-phosphate dehydrogenase in the dinoflagellate *Gonyaulax polyedra* is translationally controlled. Biochemistry.

[116] Van Dolah F.M., Leighfield T.A., Sandel H.D., Hsu C.K. (1995). Cell division in the dinoflagellate *Gambierdiscus toxicus* is phased to the diurnal cycle and accompanied by activation of the cell cycle regulatory protein, cdc2 kinase1. J. Phycol..

[117] Lin S., Zhang H., Gray M.W., Harold C.S. (2008). RNA editing in dinoflagellates and its implications for the evolutionary history of the editing machinery. RNA and DNA Editing: Molecular Mechanisms and Their Integration into Biological Systems.

[118] Lee F.W.F., Morse D., Lo S.C.L. (2009). Identification of two plastid proteins in the dinoflagellate *Alexandrium affine* that are substantially down-regulated by nitrogen-depletion. J. Proteome Res..

[119] Lee F.W.F., Lo S.C.L. (2008). The use of trizol reagent (phenol/guanidine isothiocyanate) for producing high quality two-dimensional gel electrophoretograms (2-DE) of dinoflagellates. J. Microbiol. Methods.

[120] Wang D.-Z., Zhang Y.-J., Zhang S.-F., Lin L., Hong H.-S. (2013). Quantitative proteomic analysis of cell cycle of the dinoflagellate *Prorocentrum donghaiense* (dinophyceae). PLoS ONE.

[121] Wang D.Z., Li C., Xie Z.X., Dong H.P., Lin L., Hong H.S. (2011). Homology-Driven proteomics of dinoflagellates with unsequenced genomes using MALDI-TOF/TOF and automated de novo sequencing. Evid. Based Complement. Altern. Med..

[122] Li C., Wang D.Z., Dong H.P., Xie Z.X., Hong H.S. (2012). Proteomics of a toxic dinoflagellate *Alexandrium catenella* DH01: Detection and identification of cell surface proteins using fluorescent labeling. Chin. Sci. Bull..

[123] Wang D.Z., Li C., Zhang Y., Wang Y.Y., He Z.P., Lin L., Hong H.S. (2012). Quantitative proteomic analysis of differentially expressed proteins in the toxicity-lost mutant of *Alexandrium catenella* (Dinophyceae) in the exponential phase. J. Proteom..

[124] Wang D.Z., Gao Y., Lin L., Hong H.S. (2013). Comparative proteomic analysis reveals proteins putatively involved in toxin biosynthesis in the marine dinoflagellate *Alexandrium catenella*. Mar. Drugs.

[125] Liu B., Lo S.C.-L., Matton D.P., Lang B.F., Morse D. (2012). Daily changes in the phosphoproteome of the dinoflagellate *Lingulodinium*. Protist.

[126] Wang D.Z., Lin L., Chan L.L., Hong H.S. (2009). Comparative studies of four protein preparation methods for proteomic study of the dinoflagellate *Alexandrium sp.* Using two-dimensional electrophoresis. Harmful Algae.

[127] Lee T.C.H., Ho K.C., Xu S.J.l., Lee F.W.F. (2014). Applications and challenges of proteomic technology in the study of harmful algal blooms (habs). International Conference on Biodiversity and Conservation of Wetland in South China—From Preservation to Green Development.

[128] Chan L.L., Sit W.H., Lam P.K.S., Hsieh D.P.H., Hodgkiss I.J., Wan J.M.F., Ho A.Y.T., Choi N.M.C., Wang D.Z., Dudgeon D. (2006). Identification and characterization of a “biomarker of toxicity” from the proteome of the paralytic shellfish toxin-producing dinoflagellate *Alexandrium tamarense* (Dinophyceae). Proteomics.

[129] Chan L.L., Hodgkiss I.J., Lam P.K.S., Wan J.M.F., Chou H.N., Lum J.H.K., Lo M.G.Y., Mak A.S.C., Sit W.H., Lo S.C.L. (2005). Use of two-dimensional gel electrophoresis to differentiate morphospecies of *Alexandrium minutum*, a paralytic shellfish poisoning toxin-producing dinoflagellate of harmful algal blooms. Proteomics.

[130] Wang D.Z., Lin L., Wang M.H., Li C., Hong H.S. (2012). Proteomic analysis of a toxic dinoflagellate *Alexandrium catenella* under different growth phases and conditions. Chin. Sci. Bull..

[131] Li C., Zhang Y., Xie Z.X., He Z.P., Lin L., Wang D.Z. (2013). Quantitative proteomic analysis reveals evolutionary divergence and species-specific peptides in the *Alexandrium*
*tamarense* complex (dinophyceae). J. Proteom..

[132] Snyder R.V., Gibbs P.D.L., Palacios A., Abiy L., Dickey R., Lopez J.V., Rein K.S. (2003). Polyketide synthase genes from marine dinoflagellates. Mar. Biotechnol..

[133] Tang J.Q., Li T., Yang W.D., Liu J.S., Li H.Y. (2009). Cloning and analysis of PKS gene from *Prorocentrum lima*. Acta Ecol. Sin..

[134] Ivanka T., Plamen S., Detelina B., Ivanka D.D., Rumen M., Balik D. (2012). Production of cyanobacterial toxins from two *Nostoc* species (Nostocales) and evaluation of their cytotoxicity in vitro. J. BioSci. Biotechnol..

[135] Christiansen G., Fastner J., Erhard M., Börner T., Dittmann E. (2003). Microcystin biosynthesis in planktothrix: Genes, evolution, and manipulation. J. Bacteriol..

[136] Tillett D., Dittmann E., Erhard M., von Döhren H., Börner T., Neilan B.A. (2000). Structural organization of microcystin biosynthesis in *Microcystis aeruginosa* PCC7806: An integrated peptide–polyketide synthetase system. Chem. Biol..

[137] Norte M., Padilla A., Fernández J.J. (1994). Studies on the biosynthesis of the polyether marine toxin dinophysistoxin-1 (DTX-1). Tetrahedron Lett..

[138] Macpherson G.R., Burton I.W., LeBlanc P., Walter J.A., Wright J.L.C. (2002). Studies of the biosynthesis of DTX-5a and DTX-5b by the dinoflagellate *Prorocentrum maculosum*: Regiospecificity of the putative Baeyer−villigerase and insertion of a single amino acid in a polyketide chain. J. Org. Chem..

[139] Needham J., Hu T., McLachlan J.L., Walter J.A., Wright J.L.C. (1995). Biosynthetic studies of the DSP toxin DTX-4 and an okadaic acid diol ester. J. Chem. Soc. Chem. Commun..

[140] Wright J.L.C., Hu T., McLachlan J.L., Needham J., Walter J.A. (1996). Biosynthesis of DTX-4: Confirmation of a polyketide pathway, proof of a Baeyer–villiger oxidation step, and evidence for an unusual carbon deletion process. J. Am. Chem. Soc..

[141] Daranas A.H., Fernández J.J., Norte M., Gavín J.A., Suárez-Gómez B., Souto M.L. (2004). Biosynthetic studies of the DSP toxin skeleton. Chem. Rec..

[142] Nishizawa T., Ueda A., Asayama M., Fujii K., Harada K.-I., Ochi K., Shirai M. (2000). Polyketide synthase gene coupled to the peptide synthetase module involved in the biosynthesis of the cyclic heptapeptide microcystin. J. Biochem..

[143] Kaebernick M., Neilan B.A., Börner T., Dittmann E. (2000). Light and the transcriptional response of the microcystin biosynthesis gene cluster. Appl. Environ. Microb..

